# Legal personhood and frozen embryos: implications for fertility patients and providers in post-*Roe* America

**DOI:** 10.1093/jlb/lsad006

**Published:** 2023-05-19

**Authors:** Gerard Letterie, Dov Fox

**Affiliations:** Seattle Reproductive Medicine, Seattle, Washington, USA; University of San Diego School of Law, San Diego, California, USA

**Keywords:** personhood, embryos, *Roe*, *Dobbs*, assisted reproductive technology, in vitro fertilization

## Abstract

The demise of *Roe v. Wade* has prompted some state lawmakers to try to redefine legal personhood to begin before birth and even before pregnancy. The sweeping abortion bans passed and pending in the wake of *Dobbs* pose a threat to reproductive rights that extends beyond abortion. That threat spills over into in vitro fertilization (IVF) and other assisted reproductive technologies (ART). If legislatures designate embryos as legal persons, fertility clinics will be forced to change how they manage embryos, including current standard practices such as pre-implantation genetic testing, storage of unused embryos, and the disposal of those unlikely to have reproductive potential. This essay examines the many ways in which conferring the status of persons under private and public law is likely to impact patients pursuing IVF and clinics practicing ART.

## INTRODUCTION

On June 24, 2022, the Supreme Court overturned *Roe v. Wade*, which had affirmed a constitutional right to abortion and rejected fetal personhood before birth. In abolishing the abortion right, the Court’s decision in *Dobbs v. Jackson Women’s Health Organization* also opened up space for states to confer the legal personhood status on nascent human beings as early as fertilization.^1^ Louisiana foreshadows what may be coming in many more states. A Louisiana statute on the books since 1986 defines any embryo outside of the body ‘as a juridical person’ whose destruction is categorically forbidden—not under the federal Constitution, but state law.^2^  *Dobbs* paves the way for states to go even further, prohibiting embryonic stem cell research and other reproductive practices that involve foreseeable damage to embryos.^3^ Personhood laws could bar certain uses of frozen embryos, or even their creation for purposes of assisted reproduction in a way that reflects standard-of-care practice in the United States today.^4^

Female fertility patients can avoid additional cycles of painful and risky egg retrieval by enabling providers to create more embryos than they plan to implant all at once, and then freeze the ‘spare’ embryos for future use, in case the first pregnancy doesn’t implant. Closing this option would force women to undergo multiple oocyte retrieval procedures, and could strengthen calls to mandate the ‘adoption’ of patients’ unused embryos. State laws that designate embryos as persons will also make it hard for practitioners to carry out best practices for clinical care or honor prior agreements signed before these state laws were passed. Courts could even appoint a *guardian ad litem* to negotiate fair and equitable decisions on behalf of frozen embryos. The following two scenarios are instructive.

Scenario 1: A couple has six frozen embryos in storage at their local clinic. They have two children at home and decided they no longer want to pay the $500 per month (estimated) to store their six frozen embryos. Before *Dobbs*, in every state but Louisiana, the embryos would be discarded with signed consent and agreement to that effect among the parties. After *Dobbs*, that option may not be available in many more states. Abiding by the patients’ clear wishes to discard their embryos could even open the clinic to liability for ‘wrongful death.’

Scenario 2: A man and woman divorces with four frozen embryos in storage. They disagree about what to do with them. The woman wants to implant one embryo to create a child. The man wants them destroyed. He does not want genetic parenthood forced on him. Before *Dobbs*, their disagreement could be settled in court as a function of factors including the parties’ respective interests in reproducing, or not. Now, states that ascribe personhood status to embryos will increasingly require that the embryos be given to the party who wants them implanted, even if that flies in the face of their clear agreement to the contrary.

Other scenarios are easy to imagine: For example, patients and doctors being incentivized to create and transfer multiple embryos in a single treatment, risking high risk multiple births, which are more dangerous. These cases illustrate the practical day-to-day management and decision making for any clinic that delivers assisted reproductive technologies (ART). Since the inception of in vitro fertilization (IVF) and related fertility practices, these decisions have been made according to well-defined medical guidelines that are designed to maximize patient care and outcomes. Now, the state threatens increasingly to tell fertility clinics and patients what can be done, and cannot, imposing punitive penalties for failure to comply. This essay examines the far-reaching implications that designating embryos as persons will have for the practice of ART in post-*Roe* America.^5^ We consider these implications from three critical perspectives: (i) patients; (ii) providers; and (iii) the embryo-as-shareholder.

Embryos have been described in various ways since inception of IVF in the 1970s. *Zygote*, *preembryo*, *early feto-placental unit* are among a variety of terms used to characterize life in these early stages. The political struggle for legal personhood of human embryos has transformed fetal life from a ‘biological entity into a social one’ with ‘individuality, personhood, and rights.’^6^ Before World War II, preserved fetal remains were seen as biological entities for scientific research or public display for educational value.^7^ The post-war liberalism of the 1960 and growth of fetal protectionism after *Roe* saw those same fetuses as ‘babies’ or ‘human bodies’ more worthy of burial than use.^8^ This transformation accompanied anti-abortion efforts by the religious right in the 1980s and 1990s to advance the evils of fetal pain together with photographs of late-stage fetuses.^9^ Many pro-life advocates opposed IVF in the late 1970s and early 1980s because the practice, while it aimed at creating new people, often involved the destruction of human life in the form of embryos that for one didn’t ultimately get implanted.^10^ Much of the religious right saw things differently, accepting IVF because it did not involve fetal pain.^11^ These factions came together in the 2000s and 2010s to prioritize legal recognition of fetal personhood as a means to restrict abortion access.^12^

*Dobbs* emboldens those efforts and gives them new life. The decision does not declare that embryos are constitutional persons with rights to due process and equal protection under the law. But neither does it say that they are not. And it overrules *Roe*, which had rejected such individual, personhood interests on the ground that ‘the unborn have never been recognized in the law as `persons’ or ‘accord[ed] legal rights.’^13^ That an embryo or fetus ‘represents only the potentiality of life,’ the Court declared, disqualifies that entity from having any individual interests before it is born.^14^ Its possible acquisition of such interests in the future, the Court explained, is ‘contingent upon [its] live birth.’^15^ Accordingly, not even a fully developed fetus could have any protectable interests of its own, apart from the interest in potential life that the state has in it, like it does in great works of art or endangered species.^16^

Until *Dobbs*, courts entitled frozen embryos to ‘special respect,’ on account of their ‘*potential* to become a person.’^17^ This intermediate measure of standing—‘greater than that accorded to human tissues’ like blood or hair, but less than a person—is what the Tennessee Supreme Court said embryos are owed in a 1992 divorce action between Mary Sue and Junior Davis. The former spouses agreed on all terms of the dissolution except what to do with the seven embryos that they had cryopreserved while they were married. She wanted to use them to get pregnant; he wanted them donated to a childless couple.^18^ Other states had adopted the ‘special respect’ status the Tennessee high court assigned to the frozen embryos in disposition disputes elsewhere.^19^

But *Dobbs* enhanced the legal status of potential life to the point that it justifies outright bans on abortion—until then, a fundamental constitutional right—from the moment of conception. By explicitly overruling *Roe*’s holding that abortion is a right, the *Dobbs* majority implicitly opened space to reconsider *Roe*’s separate holding that prenatal life lacks the legal status of personhood. This opening has not gone unnoticed in the states, which have variously enacted measures to ‘[f]ully recognize the human personhood of an unborn child . . . from the moment of fertilization.’^20^ Some lawmakers have suggested that such laws be interpreted to forbid interventions that involve the deliberate loss of nascent life even before pregnancy.^21^

Under current fertility medicine and technology, embryos are created either to initiate a pregnancy or freeze for future use. The availability of sensitive molecular studies has enabled fertility specialists to characterize embryos as being normal genetically or what is referred to as euploid; 1 of 2 categories of genetic abnormalities referred to as mosaicism (high vs low) or aneuploid or abnormal. Previous practices and standards of care have dictated that an abnormal embryo be discarded with essentially no implantation potential. Recent studies show an extremely low but definable likelihood of these genetically abnormal embryos resulting in a healthy live birth.^22^ This essentially calls into question the disposition of *any* embryo regardless of its genetics or appearance or predicted likelihood of ending in a healthy live birth.

The ability to freeze embryos with a high likelihood of implantation and survival has revolutionized fertility medicine, and brought with it a complexity of issues about what to do with those frozen embryos.^23^ Current technologies have success rates anywhere from 10 to 70 per cent live birth rates depending on the patient population. ^24^ Not every embryo is biologically capable of implanting and resulting in a live born baby. But it is still practically impossible to distinguish viable embryos from non-viable ones with any scientific certainty.^25^ Under all but the most extreme circumstances, the only way to prove that an embryo was non-viable is to transfer and await outcome. Thus any embryo regardless of morphology or genetic complement must be considered under these evolving concepts of personhood as resulting in a live birth.

The rationale behind the need to freeze is straightforward. Fertility medicine today aims to maximize present and future reproductive options. Clinical care seeks to create embryos for immediate use and to have a cohort available to freeze and create an inventory for future use.^26^ These future options are enabled through long-term storage facilities. Many individuals or patients who intend to create embryos to initiate a pregnancy immediately also seek to maintain others in their frozen inventory for future use.^27^ Maybe a couple is not quite prepared to move ahead with family building but is sensitive to the impact of maternal age. Or an individual woman might seek to pursue career plans, while preserving her likelihood of having children in the future. Both embryo and oocyte freezing offer options to achieve these goals. Advances in clinical care and technology have progressed to the point where embryo freezing is an essential and routine part of ART.^22^ Estimates place the number of frozen embryos at ˃1.5 million.^28^ If personhood is granted to embryos, then the laws in many more states than Louisiana are likely to bar patients and clinics from discarding them or using them for valuable medical research and clauses in the laws may preclude transporting to states with more liberal laws.^29^ In this setting the question becomes: how to manage this inventory within restrictive laws?

The recent crashes of fertility freezers illustrate the potential liability stakes that could now exist for destroying frozen embryos under the post-*Roe* regime. Hundreds of would-be parents had their dreams of biological children crushed in 2018.^30^ High-capacity storage containers failed at major medical facilities in Cleveland and San Francisco.^31^ These subzero containers are poorly regulated, no better by some accounts than kitchen appliances or farm tools.^32^ The bulk vats were developed in the 1960s to store livestock semen for breeding.^33^ Now they are used by almost five hundred fertility clinics nationwide to freeze people’s eggs and embryos at a constant −196°C. Temperatures began rising on the same unstaffed weekend that March, with remote alarms inactive.^34^ By the time lab technicians returned on Monday morning, everything inside had been thawed beyond rescue or repair. Center operators pointed the finger at defective equipment, while manufacturers blamed laboratory staff for ‘forget[ting] to refill’ the liquid nitrogen chambers in these ‘ever-dependable vessels.’^35^ After *Dobbs*, personhood laws could authorize states to sue clinics in cases like these for major liability under the doctrine of ‘wrongful death,’ characteristically but not always reserved for negligent or reckless misconduct that causes the loss of legal person.^36^

Legislatures had initially enacted wrongful death statutes to fill an untenable gap in the early common law. Liability attached only if a plaintiff survived—if he died, defendants went scot free.^37^ Wrongful death suits were designed, not to protect the life already lost, but rather to deter misconduct and compensate the victim’s survivors. Originally, recovery was allowed only for economic losses, such as funeral expenses and a loved one’s lost wages that had provided essential household income for his spouse and children. Most jurisdictions have since allowed wrongful-death plaintiffs to recover for emotional and other non-pecuniary losses of companionship and peace of mind. This allowed parents to seek redress for the wrongful death of relatives or other dependents whose heartbreaking death doesn’t set them back financially, including children whose injuries were inflicted on them, while still *in utero*, back before they were born.^38^ But this expansion invited another puzzle. ‘Wrongful death’ now afforded recovery to expecting parents whose fetuses survived a negligent injury, at least until live delivery, but not where a fetus was injured so severely that it died during pregnancy. When it came to prenatal misconduct, damages still seemed inappropriately *lower* in response to a *graver* injury.^39^

To address this apparent paradox, the majority of states expanded the cause of action again, this time to cover stillborn fetuses capable of surviving on their own. Since statutes limit its application to the death of a ‘person,’ this move required defining fetuses as persons—for the narrowly circumscribed purpose of victims who would have been parents to recover.^40^ Compensation for wrongful fetal death does not protect the lost fetus itself, or give it any rights that might be asserted against others. Instead, it speaks to the devastating loss that expectant parents endure when negligence ends their wanted pregnancy. ^41^ ‘Fetal personhood’ in this limited context did not entitle a fetus to any interests of its own—so it need not implicate the fetus’s ability to inherit property, or a woman’s right to abort it.^42^ Every court that had considered the ‘wrongful death’ of IVF embryos before *Dobbs* had rejected such claims on the ground that the term ‘person’ doesn’t apply to frozen embryos under the meaning of state law.^43^ Many cancer survivors and older fertility patients whose embryos, oocytes or sperm are negligently destroyed might also be robbed of their last chance to carry and raise a genetic child. Yet judges have so far resisted claims to permit suits for the ‘wrongful death’ of lost embryos like they have for post-viability fetuses. After *Dobbs*, liability risks could attach for any damage to embryos in transporting or receiving from one clinic to the other, or if spilling culture media in the lab and losing several embryos or if there is active decision making on the part of an individual or couple to discard the embryo. Added to this is the complexity of insurance coverage for everything from medical malpractice to criminal abandonment.^44^

Options that have been considered as possible solutions are embryo donation and restricting the number of eggs, or oocytes, that are inseminated and thus the number of embryos in storage. Donation has been talked about as a win–win (excess embryos ``adopted'' by those interested in pregnancy) but a relatively low uptake. In a recent survey only 15 per cent of patients are willing to consider embryo donation.^45^ Would-be recipients are generally reluctant to use embryos that were generated from an infertile couple where the embryos’ implantation potential is unclear.^46^

Though appealing in concept, the reality of embryo donation is far more complicated. Three perspectives influence this option. From the perspective of patients who are interested in donating embryos, key are issues related to identification and disclosure of the donating families. The term of *anonymous donors* in any context has been replaced by the term *non-identified donors*.^47^ This change in language reflects the source of concern among would-be donors and relates to the inability to assure anonymity with the prevalence of nonmedical/direct-to-consumer and social media and networking.^48^ Other issues that prompt couples to decline embryo donation relates to the simple fact that many families take a narrow view of having their embryos *at large* with no control over their destiny.

From the standpoint of recipient families, donated embryos are derived from patients undergoing IVF for reasons relating to infertility and thus have attached to them a variable success rate depending on the clinical indications for the IVF cycle.^49^ In addition to this, the ‘de-selected’ embryos that remain in inventory are those of lower implantation potential from the cohort derived from the IVF cycle (the more viable embryos usually have been transferred).^50^ This leads to a lower likelihood of success for the recipient family. An urgency and need to move forward quickly usually prompts patients that have exhausted other family building options to move forward with the most expeditious next step.^51^

From the standpoint of providers, many of the patients have not been adequately screened prior to the generation of these stored embryos.^52^ These embryos may be 15 years old and frozen at a time prior to the extensive infectious screening now in place. The US Food and Drug Administration (FDA), American Association of Tissue Banks, US Centers for Disease Control and Prevention (CDC), and American Society for Reproductive Medicine (ASRM) have developed extensive safeguards for the optimal and safe storage and donation of any tissue embryos included.^53^ A waiver can be attached with the following explicit statement: ‘WARNING: NOT EVALUATED FOR INFECTIOUS SUBSTANCES’. The US FDA, American Association of Tissue Banks, US CDC, and ASRM have developed extensive safeguards for the optimal and safe storage and donation of any tissue embryos included.

The second option of restricting the number of oocytes inseminated has been explored particularly in Italy as an example of government regulation of ART gone awry. The passage of Law 40/2004 in Italy, which aims to prevent the ‘loss of *any* early human embryo,’ dramatically affected the manner assisted reproduction was conducted there.^54^ A recent attempt to modify this highly restrictive legislation failed to gain popular support and was defeated in a 2005 referendum. It had the unintended impact of forcing couples to move their care to other countries.^55^ The idea that some states are advancing after *Dobbs* is a variation of this theme to restrict the number of oocytes inseminated and thus reduce the number of embryos to contend with. As suggested by the Italian experience, it is a flawed process divorced from the patient’s interest of best outcomes in the shortest period with maximum future options.^56^ The inefficiency of the process is instructive. IVF seeks to maximize the number of embryos from each cycle to assure optimal present and future outcomes. This need is predicated on the unreliable and unpredictable outcomes regarding sperm-oocyte interaction, fertilization, and embryo development.^57^ Added to this is an inability to identify which oocytes will yield quality embryos. Absent that, insemination of all oocytes offers the most informative and efficient path forward. For example, perfect ‘looking’ oocytes will result in a fertilization rate of only ⁓80 per cent and embryo development of 30 per cent under the best circumstances.^58^ These outcomes can be even lower depending on clinical circumstances such as a maternal age beyond the age of 38 that will markedly decrease the number of oocytes available.^59^

This ‘limited insemination’ option put forth also frustrates another key element to contemporary IVF practices, namely generating sufficient number of embryos to freeze for future use. Cryotechnology has enabled patients to build an inventory of embryos frequently more than what they will ever use.^60^ These well-defined goals and definition of best outcomes may pose one of the greatest conflicts with the *Dobbs* decision: how to manage embryos unused embryos in a system where the option to discard is no longer available.^61^ The goal of the IVF process sets up a conflict and possible liability if laws restrict the options for management.^62^

The relationship between patients and their providers is fundamental to high-quality care.^63^ The patient–provider decision process has been upended where the state grants rights to the embryo that supersede the interests of patients and physician guidance. This insertion of the state runs counter to the cherished relationship providers share with patients. The elements of a healthy provider–patient relationship include (i) evidence-based recommendations for decision making within the doctor–patient relationship; (ii) joint doctor–patient advocacy for best care and clear communication among all parties; and (iii) privacy, confidentiality, trust and a safe zone for planning effective care to reach decisions on best outcomes and patient interests.^64^ In the realm of IVF, the decision making, and strategizing is especially complex. It involves embryos with the assumption that decisions regarding the embryos are made with the patients representing their interests in relationship to the embryos.^65^ Decision making between patient and provider is an extremely nuanced exchange.^66^ Intrinsic to this process is faith on the part of the patient that a provider will make the decision in their best interest based on the best evidence to ensure the best outcome. State mandates about management of reproductive options may force decisions that neither provider nor patient want and are not in the patients’ best interest.

The point of the IVF process is to create the circumstances these laws are intended to restrict: namely, to fertilize all oocytes and create as many embryos as clinically safe and effective. These restrictions negatively impact a range of goals beyond treating infertility. These include genetic screening of embryos as a form of very early prenatal diagnosis; fertility preservation and the empowerment of women; oncofertility and the option of cancer patients to preserve future fertility in the face of cytotoxic chemotherapy and its impact on fertility and the fertility infrastructures on which much of the LGBTQIA+ community (LGBTQIA+ is an abbreviation for lesbian, gay, bisexual, transgender, queer or questioning, intersex, asexual, and more.) looks for their family building options.^53^ The argument is that the entire delivery of care within the infertility sector will be impossible to execute on if the laws currently in place or proposed are enforced.^54^ Enforcement will ignore the inexactitudes at play in defining embryo viability and how to navigate within these restrictions.^55^ State laws could bar practitioners from developing a treatment plan that would be in the patient’s best interest but constrained by law. *Dobbs* could restrict clinics’ ability to treat patients to provide them with quality fertility care.

The threat is two-fold. First, is the erosion of the doctor–patient relationship and impact on trust based on interference with clinical decisions in patients’ best interest. The second threat is related: the risk for liability, including possible criminal prosecution for provider and patient alike. When it comes to the liability threat, this could involve not just civil penalties like malpractice but criminal sanctions from fines to prison. This shadow and threat may constrain options considered best treatment for a patient. Providers could be conflicted: risk prosecution or abide by legal constraints and the safety zone that this compliance may render. Lawsuits involving IVF centers are infrequent, but the era post-*Dobbs* may change both the frequency and the penalties paid.^67^ The attention post-*Dobbs* has largely centered on its impact on abortion access and penalties to both providers and patients should violations ensue. But state policies could affect everything from how miscarriages are managed and IVF.^68^ At issue in the setting of ART is how restrictive laws that ban or severely limit abortion with penalties attached for violators will impact IVF. The definition on which limits for IVF could turn is how the laws define when life begins, and if under state laws, will embryos have legal protections of personhood before transfer. If they do, conducting IVF could become much more complicated in those states. Unresolved questions about the thousands of IVF embryos that are currently sitting in freezers there would loom.^69^

Placing these possibilities in a brief historical context may be of value to gain insight into possible trends ahead. IVF restriction after *Dobbs* could follow a path like the early efforts by anti-abortion legislatures to restrict abortion services. Much of this legislation prior to *Dobbs* while not eliminating abortion services resulted in restrictive rules and regulations intended to make practice of abortion services complicated, expensive and for smaller clinics unattainable.^70^ For example, in Texas regulations were passed to require centers performing abortions to meet criteria applied to surgical centers.^71^ Fulfillment would mean as examples expanding hallway width and adding expensive anesthesia equipment. Severe penalties were enforced for noncompliance.^72^ A similar path could be envisioned at this early stage where regulations may restrict common IVF procedures such as preimplantation genetic testing limiting but not eliminating (at least at this time) options available that would assure best outcomes but not clearly (at this time) eliminating the IVF options.^73^ Total bans are unlikely soon. But hastily prepared laws with vague language could have unintended consequences for providers and patients alike.

Expanded liability and risks of prosecution are eroding patient centric care and instead shifts provider focus to a defensive posture. Recent events have brought risks of criminal prosecution adding a new and alarming layer to an already complex process. Criminal liability and loss of licensure now add to concerns about medical malpractice.^74^ This transition from medical malpractice to criminal charges is increasing in frequency, an unlikely event in the past. In addition to past cases, a recent conviction against a Vanderbilt University nurse of two felonies for a fatal drug error highlights the position that courts are taking for errors that result in fatalities.^75^ The prospect of criminal indictment should give pause to any practitioner in the ART space but added to this is possible lack of insurance coverage for these claims. Medical malpractice policies do not cover criminal misconduct.^76^ Accidents happen in any clinical setting. In IVF, embryos may be unintentionally damaged or discarded. But the implications for error in this setting post-*Dobbs* changes the calculus and imposes a far greater risk, to say nothing of actual charges that sound in criminal negligence.^77^

The risk of criminality is clear in recent state laws. A North Dakota law currently defines murder as when one ‘[i]intentionally or knowingly causes the death of another human being’ or when one ‘[c]auses the death of another human being under circumstances manifesting extreme indifference to the value of human life.’ Assuming, then, that life begins at conception, doctors who administer IVF would apparently be acting with the intent or at least with indifference to the lives of the multiple embryos with unknown viability, some which could result in a live birth and others that simply would not survive. These risks could also extend to other staff such as nurse, administrators, hospital staff, and other medical assistants, who could be guilty of accomplice crimes, including conspiracy to murder. Women and men who hope to become parents through IVF could also be criminally liable.

Criminal penalties have not yet been defined, but the language of some bills advanced in state legislature is cause for alarm on the part of practitioners and patients alike. Louisiana lawmakers advanced a bill that would grant constitutional rights to ‘all unborn children from the moment of fertilization’ and classify abortion as homicide. The bill defines personhood as beginning from the moment of fertilization that would subject people to murder prosecutions, punishable by life without parole, for having abortions.

A natural end point of these threats to care is this: practitioners may find themselves in a position fraught with liability on two fronts: duty bound to make decisions in a patient’s best interest but legally held responsible for a decision that may honor the law but betray a patient trust. Put differently, practitioners may have a conflict between using the highest clinical standards for patient care according to the principles of beneficence and non-malfeasance or abide by strict laws that run counter to these principles. Patients may find themselves accountable to laws that are against their interests; prior commitments and contracts and exacerbate the vulnerability that these patients carry with them. Personhood laws would pull fertility doctors between opposing obligations—their commitment to treat patients with sound care, and their obedience to the law. This crisis of conscience will exact a deep psychological toll on clinicians and diminish the trust patients have in them to put their medical interests first. This conflict threatens to arrest and upend 50 years of bioethics progress for the well-being of patients in fertility science, medicine, and technology.

## Footnotes

1 I. Glenn Cohen, Judith Daar & Eli Y. Adashi, Opinion, *What the Supreme Court’s Abortion Reversal Means for In Vitro Fertilization*, Bos. Globe (June 30, 2022, 3:15 AM), https://www.bostonglobe.com/2022/06/30/opinion/what-supreme-courts-abortion-reversal-means-vitrofertilization/ [https://perma.cc/3KXG-M487].

2 La. Rev. Stat. § 9:121 (West 2021).

3
*See Doe v. Obama*, 631 F.3d 157, 160 (4th Cir. 2011); Sarah Zhang, *Can Lost Embryos Give Rise to a Wrongful-Death Suit?*, Atlantic (Apr. 5, 2018), https://www.theatlantic.com/health/archive/2018/04/fertility-clinicembryos/557258/ [https://perma.cc/VH39-2N88].

4
*See* Steven R. Morrison, *Personhood Amendments After* Whole Woman’s Health v. Hellerstedt, 67 Case w. Res. L. Rev. 447, 453–57 (2016).

5
*See* Henry T. Greely, *The Death of Roe and the Future of Ex Vivo Embryos*, 9 J. L. & Biosciences 1 (2022).

6 Sara Dubow, *Ourselves Unborn: A History of the Fetus in Modern America* 41 (2010).

7
*Id.* at 171.

8
*Id*. at 38–44.

9
Sara Dubow, Ourselves Unborn: A History of the Fetus in Modern America 157 (2010).

10 Daniel K. Williams, *Defenders of the Unborn: The Pro-Life Movement Before Roe v. Wade* 266 (2016).

11
*Id*.

12
Mary Ziegler, Abortion and the Law in America 184 (2020).

13
*Roe v. Wade*, 410 U.S. 113, 161–62 (1973).

14
*Id*. at 156.

15
*Id*. at 162.

16
*Id.*

17
*Davis v. Davis*, 842 S.W.2d 588, 602 (Tenn. 1992) (emphasis added).

18
*Id*. at 596–97.

19
*See, eg*, *Jeter v. Mayo Clinic Ariz*., 121 P.3d 1256, 1266–68 (Ariz. Ct. App. 2005); *McQueen v. Gadberry*, 507 S.W.3d 127, 148–49 (Mo. Ct. App. 2016).

20
*Eg*, H.B. 813, 2022 Leg., Reg. Sess. (La. 2022); H.R. 4327, 58th Leg., 2d Reg. Sess. (2022 Okla.); Utah Code Ann. § 76–7-301 (West 2021); Ky. Rev. Stat. Ann. § 311.715 (West 2022).

21
*See, eg*, Ashton Pittman, *Mississippi Leaders Supported 2011 Initiative Targeting Abortion, Contraception, IVF*, Miss. Free Press (May 16, 2022), https://www.mississippifreepress.org/23762/mississippileaders-supported-2011-initiative-targeting-abortion-contraception-ivf [https://perma.cc/VFN6-ZASD]; Tessa Weinberg, *‘Anything’s on the Table’: Missouri Legislature May Revisit Contraceptive Limits Post-*Roe, MO. Independent (May 20, 2022, 9:00 AM), https://missouriindependent.com/2022/05/20/anythings-on-the-tablemissouri-legislature-may-revisit-contraceptive-limits-post-roe/ [https://perma.cc/4DDZ-HVXH]; Guilia Carbonaro, Roe v. Wade *Being Overturned Could See IVF Banned in at Least 30 States*, Newsweek (June 14, 2022, 9:16 AM), https://www.newsweek.com/roe-v-wade-being-overturned-ivf-banned-30-states-1715576 [https://perma.cc/87MUGGFN].

22
*See* Norbert Gleicher, Pasquale Patrizio & Ali Brivanlou, *Preimplantation Genetic Testing for Aneuploidy – A Castle Built on Sand,* 27 Trends Molecular Med. 731 (2021), https://pubmed.ncbi.nlm.nih.gov/33446425/.

23
*See* P.R. Koninckx & P. Schotsmans, *Frozen Embryos: Too Cold to Touch? Spare Embryos: Symbols of*  *Respect for Humanity and Freezing in the Pronuclear Stage*, 11 Hum. Reprod. 1841 (1996), https://academic.oup.com/humrep/article/11/9/1841/615962.

24
*See*  Ctrs. For Disease Control, Art Success Rates, https://www.cdc.gov/art/artdata/index.html [https://perma.cc/8UVE-67L6].

25
*See* David K. Gardner, et al. *Diagnosis of Human Preimplantation Embryo Viability,* 21 Hum. Reprod. Update 727 (2015), https://pubmed.ncbi.nlm.nih.gov/25567750/.

26
*See* Laura Francesca Rienzi, *Perspectives in Gamete and Embryo Cryopreservation,* 36 Seminars in Reprod. Med. 253 (2019), https://pubmed.ncbi.nlm.nih.gov/30947341/.

27
*See* S. Canosa et al., *The Effect of Extended Cryo-Storage Following Vitrification on Embryo Competence: A Systematic Review and Meta-Analysis,* 39 J. Assisted Reprod. Genetics 873, https://pubmed.ncbi.nlm.nih.gov/35119549/.

28
*See* Gerard Letterie, In re: The Disposition of Frozen Embryos: 2022, 177 Fertility & Sterility 477 (2022), https://pubmed.ncbi.nlm.nih.gov/35131103/.

29
*See* David Badash, *‘From the Moment of Fertilization’: Louisiana Advances Bill Criminalizing Abortion as Homicide – Women, Doctors Could be Jailed,*  Alternet (May 6, 2022), https://www.alternet.org/2022/05/louisiana-house-abortion/.

30
*See* Ariana Eunjung Cha, *These Would-be Parents’ Embryos Were Lost. Now They’re Grieving—And Suing*, Wash. Post (Aug. 24, 2018), https://www.washingtonpost.com/national/health-science/these-would-beparents-embryos-were-lost-now-theyre-grieving—and-suing/2018/08/24/57040ab0-733c-11e8-805c4b67019fcfe4_story.html?noredirect=on&utm_term=.80e17d-f7e769.

31
*See* Natalie Lampert, Their Embryos Were Destroyed: Now They Mourn the Children They’ll Never Have, The Guardian (May 13, 2018), https://www.theguardian.com/lifeandstyle/2018-/may/-13/their-embryoswere-destroyed-now-they-mourn-the-children-theyll-never-have.

32
*See* Kayla Webley Adler, *When Your Dreams of Motherhood Are Destroyed*, Marie Claire (Oct. 1, 2018), https://www.marieclaire.com/health-fitness/a23327231/egg-freezing-emryos-lack-of-regulation/.

33
*See* Amy Goldstein, *Fertility Clinic Informs Hundreds of Patients Their Eggs May Have Been Damaged*, Wash. Post (Mar. 11, 2018), https://www.washingtonpost.com/national/healthscience/fertility-clinic-informshundreds-of-patients-their-eggs-may-be-damaged/2018/03/11/b605ea82-2536-11e8-b79df3d931db7f68_story.html?utm_term=.1155a73dbbec.

34
*See* Rich Gardella & Erika Edwards, *Heartbreak, anxiety, lawsuits: The egg-freezing disaster a year later*, Nbc News (Mar. 4, 2019), https://www.nbcnews.com/news/all/heartbreak-anxietylawsuits-egg-freezingdisaster-year-later-n978891.

35 Mitchel C. Schiewe et al., *Comprehensive Assessment of Cryogenic Storage Risk and Quality*  *Management Concerns: Best Practice Guidelines for ART Labs*, 36 J. Assisted Reprod. & Genetics 5, 5 (2019); *see also* Zahava P. Michaelson et al., *Early Detection of Cryostorage Tank Failure Using a Weight-based Monitoring System*, 36. Assisted Reprod & Genetics 655 (2019).

^36^

*See* Gerard Letterie, *In re: The Disposition of Frozen Embryos: 2022,* 177 Fertility & Sterility 477 (2022), https://pubmed.ncbi.nlm.nih.gov/35131103/.

37
*See* Wex S. Malone, *The Genesis of Wrongful Death*, 17 Stan. L. Rev. 1043, 1062–66 (1965).

38
*See*  William Prosser & W. Page Keeton, The Law of Torts § 127, at 945 (5th ed. 1984).

39
*See*  Dov Fox, Birth Rights and Wrongs 48–49 (2019); Dov Fox, *Reproductive Negligence*, 117 Columbia Law Review 149, 218 (2017).

40
*See* Dov Fox, *Interest Creep*, 82 George Washington Law Review 273, 279 (2014).

41
*See* Dov Fox, *Redressing Future Intangible Losses*, 69 DePaul Law Review 419, 430 (2019).

42
*See, eg*, Carranza v. United States, 267 P.3d 912 (Utah 2011); Summerfield v. Superior Court, 698 P.2d 712, 715, 724 (Ariz. 1985).

43
*See* McClain v. Univ. of Mich. Bd. of Regents, 665 N.W.2d 484, 486 (Mich. Ct. App. 2003); Miccolis v. Amica Mut. Ins. Co., 587 A.2d 67, 71 (1991); Gentry v. Gilmore, 613 So.2d 1241, 1244 (Ala. 1993); Jeter v. Mayo Clinic Ariz., 121 P.3d 1256, 1261-62 (Ariz. Ct. App. 2005); Miller v. Am. Infertility Grp. of Ill., 897 N.E.2d 837, 839-40 (Ill. App. Ct. 2008).

44
*See* Jennifer F. Kawwass et al., *Embryo Donation: National Trends and Outcomes, 2000–2013,* 215 Amer. J. Obstetrics Gynecology 747.e1, https://pubmed.ncbi.nlm.nih.gov/27393270/.

45
*See* Alison E. Zimon, et al., *Embryo Donation: Survey of In-Vitro Fertilization (IVF) Patients and Randomized Trial of Complimentary Counseling,* 14 Plos One e0221149, https://pubmed.ncbi.nlm.nih.gov/31415660/.

46
*See* V. Jadva, et al., *Sperm and Oocyte Donors’ Experiences of Anonymous Donation and Subsequent*  *Contact with Their Donor Offspring,* 26 Human Reprod. 638 (2011), https://pubmed.ncbi.nlm.nih.gov/21177310/.

47
*See* Julinda Lee, *Embryo Donation: A Review,* 82 Acta Obstectrica et Gynecologica Scandinavica 991 (2003), https://pubmed.ncbi.nlm.nih.gov/14616271/.

48
*See* Prac. Comm. of the Amer. Soc. for Reprod. Med. and the Prac. Comm. for the Soc. for Assisted Reprod. Tech., *Guidance Regarding Gamete and Embryo Donation*, 115 Fertility & Sterility 1395 (2021), https://pubmed.ncbi.nlm.nih.gov/33838871/ [hereinafter *Gamete and Embryo Donation Guidance*].

49
*See* Guido Pennings, et al., *Attitudes of Sperm Donors Towards Offspring, Identity Release and Extended Genetic Screening*, 43 Reprod. Biomed. Online 700 (2021), https://pubmed.ncbi.nlm.nih.gov/34412975/.

50
*See* K Wånggren, et al., *Attitudes Towards Embryo Donation Among Infertile Couples with Frozen Embryos*, 28 Human Reprod. 2432 (2013), https://pubmed.ncbi.nlm.nih.gov/23756704?.

51
*See* Harry Hatasaka, *An Efficient Infertility Evaluation*, 54 Clinical Obstetrics Gynecology 644 (2011), https://pubmed.ncbi.nlm.nih.gov/22031254/.

52
*See Gamete and Embryo Donation Guidance*, *supra* note 39.

53
*See*  Food & Drug Admin., Guidance for Industry: Eligibility Determination for Donors of Human Cells, Tissues, and Cellular and Tissue-Based Products (Hct/Ps) (2007), https://www.fda.gov/media/73072/download [https://perma.cc/5FGB-WJWF].

54
*See* Giuseppe Benagiano & Luca Gianaroli*, The New Italian IVF Legislation,* 9 Reprod. Biomed. Online 117-118 (2004), https://pubmed.ncbi.nlm.nih.gov/15333237/.

55
*See* Mark V. Sauer, *Italian Law 40/2004: A View from the ‘Wild West,’* 12 Reprod. Biomed. Online 8 (2006), https://pubmed.ncbi.nlm.nih.gov/16454924/.

56
*See* Dmitry Nikiforov et al., *Human Oocyte Morphology and Outcomes of Infertility Treatment: a Systematic Review*, Reprod. Sci. (2021), https://pubmed.ncbi.nlm.nih.gov/34816375/.

57
*See* Jose Buratini et al., *Maternal Age Affects the Relationship of Basal FSH and Anti-Müllerian Hormone Concentrations with Post-ICSI/IVF Live Birth*, 42 Reprod. Biomed. Online 748 (2021), https://pubmed.ncbi.nlm.nih.gov/33653653/.

58
*See* Marine Poulain et al., *Impact of Ovarian Yield-Number of Total and Mature Oocytes Per Antral Follicular Count-On Live Birth Occurrence After IVF Treatment*, 8 Frontiers in Med. (2021), https://pubmed.ncbi.nlm.nih.gov/34504852.

59
*See* Natalie M. Crawford & Anne Z. Steiner, *Age-related Infertility*, 42 Obstetrics Gynecology Clinics N. Amer. 15 (2015), https://pubmed.ncbi.nlm.nih.gov/25681837/.

60
*See* Adam S. Cifu, *Long-term Physician-Patient Relationships—Persevering in a Practice*, 179 Jama Internal Med. 141 (2019), https://pubmed.ncbi.nlm.nih.gov/30508031/.

61
*See* Selena E. Ortiz & Meredith B. Rosenthal, *Medical Marketing, Trust, and the Patient-Physician Relationship*, 32 Jama 40 (2019), https://pubmed.ncbi.nlm.nih.gov/30620354/.

62
*See id*.

63
*See id.*

64
*See* Jacquelin Forsey et al., *The Basic Science of Patient-Physician Communication: A Critical Scoping Review*, 96 Academic Med. J. Assoc. Amer. Med. Colleges S109 (2021), https://pubmed.ncbi.nlm.nih.gov/34348382/.

65
*See* Catherine A. McMahon & Douglas M. Saunders*, Attitudes of Couples with Stored Frozen Embryos Toward Conditional Embryo Donation*, 91 Fertility Sterility 140 (2009), https://pubmed.ncbi.nlm.nih.gov/18053994/.

66
*See ACOG Committee Opinion No. 587: Effective Patient-physician Communication*, 123 Obstetrics Gynecology 389 (2014), https://pubmed.ncbi.nlm.nih.gov/24451677/.

67
*See* Gerard Letterie, *Outcomes of Medical Malpractice Claims in Assisted Reproductive Technology over a 10-year Period from a Single Carrier*, 34 J. Assisted Reprod. & Genetics 459 (2017), https://pubmed.ncbi.nlm.nih.gov/28190212/.

68
*See* Aria Bendix, *States Say Abortion Bans Do not Affect IVF. Providers and Lawyers Are Worried Anyway*, Nbc News (June 29, 2002, 9:56 AM), https://www.nbcnews.com/health/health-news/states-say-abortionbans-dont-affect-ivf-providers-lawyers-worry-rcna35556 [https://perma.cc/GLJ5-U9MB].

69
*See* Jan Hoffman, *Infertility Patients and Doctors Fear Abortion Bans Could Restrict I.V.F.,*  N.Y. Times, (July 6, 2022), https://www.nytimes.com/2022/07/05/health/ivf-embryos-roe-dobbs.html [https://perma.cc/F6UPQ7WC].

70
*See* Planned Parenthood Action Fund, *What Are TRAP Laws?*, https://www.plannedparenthoodaction.org/issues/abortion/types-attacks/trap-laws [https://perma.cc/5MDB-PCAX].

71
*See*  Tex. Health & Safety Code Ann. §§ 245.001–245.025 (West 2021), https://statutes.capitol.texas.gov/Docs/HS/htm/HS.245.htm.

72
*See* James Studnicki et al., *Doctors Who Perform Abortions: Their Characteristics and Patterns of Holding and Using Hospital Privileges*, 6 Health Svcs. Res. & Managerial Epidemiology (2019), https://pubmed.ncbi.nlm.nih.gov/31020009/.

73
*See* Carey Goldberg, *Abortion Ruling Clouds Future for In Vitro Fertility Patients,*  Bloomberg (June 29, 2022, 2:00 AM), https://www.bloomberg.com/news/articles/2022-06-29/roe-v-wade-decision-clouds-future-forin-vitro-fertility-patients.

74
*See* Julia B. Berman & Guohua Li, *Characteristics of criminal cases against physicians charged with opioid-related offenses reported in the US news media*, 1995–2019, 7 Injury Epidemiology 1 (2020), https://pubmed.ncbi.nlm.nih.gov/32998773/.

75
*See* Brett Kelman, *In Nurse’s Trial, Witness Says Hospital Bears ‘Heavy’ Responsibility for Patient Death*, NPR (Mar. 24, 2022, 5:00 PM), https://www.npr.org/sections/health-shots/2022/03/24/1088397359/innurses-trial-witness-says-hospital-bears-heavy-responsibility-for-patient-dea [https://perma.cc/XYK3-XW8Y].

76
*See* M.M. Reidenberg & O. Willis, *Prosecution of Physicians for Prescribing Opioids to Patients*, 81 Clinical Pharmacology & Therapeutics 903 (2007), https://pubmed.ncbi.nlm.nih.gov/17329989/.

77
*See* Natasha Kay et al., *Should doctors who make clinical errors be charged with manslaughter? A survey of medical professionals and members of the public*, 48 Medicine, Science, and the Law 317 (2008), https://pubmed.ncbi.nlm.nih.gov/19051669/.

